# Risk factors for death in patients with severe asthma*

**DOI:** 10.1590/S1806-37132014000400003

**Published:** 2014

**Authors:** Andréia Guedes Oliva Fernandes, Carolina Souza-Machado, Renata Conceição Pereira Coelho, Priscila Abreu Franco, Renata Miranda Esquivel, Adelmir Souza-Machado, Álvaro Augusto Cruz

**Affiliations:** Programa para o Controle da Asma na Bahia - ProAR, Bahia State Asthma Control Program - Salvador, Brazil; School of Nursing. Federal University of Bahia, Salvador, Brazil; Federal University of Bahia School of Medicine, Salvador, Brazil; Bahia School of Medicine and Public Health, Salvador, Brazil; Center of Excellence in Asthma, Federal University of Bahia, Salvador, Brazil; Institute of Health Sciences, Federal University of Bahia; and Coordinator. Programa para o Controle da Asma na Bahia - ProAR, Bahia State Asthma Control Program - Salvador, Brazil; Federal University of Bahia School of Medicine, Salvador, Brazil

**Keywords:** Asthma/mortality, Asthma/therapy, Risk factors

## Abstract

**OBJECTIVE:**

To identify risk factors for death among patients with severe asthma.

**METHODS:**

This was a nested case-control study. Among the patients with severe asthma treated between December of 2002 and December of 2010 at the Central Referral Outpatient Clinic of the Bahia State Asthma Control Program, in the city of Salvador, Brazil, we selected all those who died, as well as selecting other patients with severe asthma to be used as controls (at a ratio of 1:4). Data were collected from the medical charts of the patients, home visit reports, and death certificates.

**RESULTS:**

We selected 58 cases of deaths and 232 control cases. Most of the deaths were attributed to respiratory causes and occurred within a health care facility. Advanced age, unemployment, rhinitis, symptoms of gastroesophageal reflux disease, long-standing asthma, and persistent airflow obstruction were common features in both groups. Multivariate analysis showed that male gender, FEV_1_ pre-bronchodilator < 60% of predicted, and the lack of control of asthma symptoms were significantly and independently associated with mortality in this sample of patients with severe asthma.

**CONCLUSIONS:**

In this cohort of outpatients with severe asthma, the deaths occurred predominantly due to respiratory causes and within a health care facility. Lack of asthma control and male gender were risk factors for mortality.

## Introduction

Asthma is a common chronic respiratory disease that has a substantial impact on morbidity and mortality worldwide. It is estimated that 10% of all individuals with asthma have the severe form of the disease, which has negative economic and social effects, resulting in a disproportional burden in terms of the utilization of health care services, as well as impaired quality of life and immeasurable human suffering due to recurrent episodes of asphyxiation.^(^ 1 ^,^ 2 ^)^

Asthma mortality rates have not increased in parallel with increases in the prevalence of the disease.^(^ 1 ^)^ Studies have shown that countries in which the number of deaths from asthma has decreased or remained stable are those that have adopted certain strategies aimed at controlling the disease: focusing on early diagnosis^(^ 3 ^,^ 4 ^)^; providing asthma treatment at primary health care facilities^(^ 4 ^-^ 6 ^)^; expanding/simplifying access to health care services^(^ 5 ^,^ 7 ^)^; developing educational programs and activities aimed at asthma control^(^ 3 ^,^ 4 ^)^; and providing appropriate training for health care professionals.^(^ 3 ^)^

A better understanding of the risk factors for mortality in asthma will allow the development of measures that are more effective in preventing deaths from the disease.^(^ 8 ^)^ The known risk factors for death from asthma as are follows: greater asthma severity^(^ 7 ^,^ 9 ^)^; a lack of continuity in medical visits^(^ 8 ^,^ 10 ^)^; adverse socioeconomic or psychosocial conditions^(^ 7 ^,^ 9 ^,^ 11 ^)^; and poor practices in the approach to treating the disease (lack of access to effective therapies, non-adherence to treatment, and inadequate management of the symptoms).

The objective of this study was to identify factors associated with mortality among asthma outpatients followed for nearly 10 years via an asthma control program in Brazil.^(^ 12 ^)^

## Methods

### Study design, sample, and site 

This was a nested case-control study. The study sample comprised 58 cases of death from asthma and 232 cases of severe asthma not resulting in death. All of the patients evaluated had been treated at the at the Central Referral Outpatient Clinic of the *Programa para o Controle da Asma na Bahia* (ProAR, Bahia State Asthma Control Program), in the city of Salvador, Brazil, between December of 2002 and December of 2010. The study was approved by the local research ethics committee. 

The main goal of the ProAR is to coordinate activities related to the prevention of and treatment of patients with severe asthma, within the context of the Brazilian *Sistema Único de Saúde* (SUS, Unified Health Care System). Patients enrolled in the program have access, on a quarterly basis, to free medication and consultations with a multidisciplinary health care team, as well as to asthma training and education sessions. These interventions are aimed at achieving and maintaining good asthma control.^(^ 13 ^)^

At ProAR enrollment, a pulmonologist made the diagnosis of asthma, as well as classifying the severity of each case of asthma, on the basis of the symptoms identified and the measurement of PEF. The diagnosis of asthma and the classification of its severity were in accordance with the criteria established in the Global Initiative for Asthma.^(^ 14 ^)^ The level of asthma control was determined with the Portuguese-language version of the six-item Asthma Control Questionnaire, validated for use in Brazil,^(^ 15 ^)^ which evaluates asthma symptoms and rescue bronchodilator use in the last seven days. The cut-off score found to be the most accurate in identifying uncontrolled asthma is 1.5, patients with an average score ≥ 1.5 being less likely to have achieved good asthma control.^(^ 15 ^)^

### Identification of cases

We evaluated all records of patients enrolled in the ProAR and clinically diagnosed with severe asthma, including only those who had been followed by a multidisciplinary team and had used an inhaled corticosteroid on a regular basis for at least three months. From among those patients, we identified those who evolved to death during the study period. 

We identified deaths from asthma by reviewing the ProAR patient charts for the study period. Deaths were recorded when reported by family members or when revealed by an active search for a patient who had failed to appear for scheduled visits for six months or more. 

Within the population studied, there were 62 deaths. For 8 of those deaths, the patient charts were incomplete and it was necessary to conduct home visits in attempts to obtain copies of the death certificates. In 4 cases, family members or neighbors confirmed the deaths. In the 4 remaining cases, the researchers were advised to avoid attempting to visit the residences, because they were located in neighborhoods that are considered to be high-crime areas. 

Copies of the death certificates were filed with the respective patient medical charts. When no death certificate was available, we created a provisory document, containing the pertinent information (date, time, place, and underlying cause of death), and delivered it to the Health Information Board of the Bahia State Department of Health, with a copy to the Health Information Council of the Salvador Municipal Health Department. 

### Identification of the controls

We selected additional patients with severe asthma to serve as controls. The controls were chosen at random from among all of the ProAR patients with severe asthma who did not evolve to death during the study period. We made the selection using a database of all existing ProAR patient charts, in the program Microsoft Excel 2010.

### Data collection

The study sample was stratified by age bracket: 10-30 years; 31-50 years; and > 50 years. Each randomly selected control was also assigned to the appropriate age bracket. We selected 4 controls for every death, and controls were paired with deaths by the year of the last medical visit. 

Data were collected from home visit reports, death certificates, and the medical charts on file at the health care facility. The medical charts comprised structured printouts and were organized as follows: the follow-up report of the clinical history of the patient since the previous medical visit; reports of consultations with the nursing staff, medical staff, psychologists, and social workers; records related to the enrollment of the patient in the program; copies of all examination and test results; and records of the medications dispensed by the pharmacy. The charts were systematically updated at every routine, quarterly visit.

We analyzed the following: sociodemographic data (age, gender, employment status, level of education, and place of birth); clinical data (time since enrollment in the program, number of hospital admissions, number of emergency room visits, duration of daily pulse therapy with an oral corticosteroid [≤ or > 3 days], number of asthma exacerbations, duration of the disease, and the level of asthma symptom control at the most recent ProAR evaluation); family history of asthma; smoking status; results of tests (pulmonary function tests and allergy tests); medications dispensed by the pharmacy and patient adherence to the pharmacological treatment regimen; and data related to the death (date, time, and place, as well as the underlying and contributing causes).

### Statistical analysis

The data were analyzed with the Statistical Package for the Social Sciences, version 17.0 (SPSS Inc., Chicago, IL, USA). Categorical variables are presented as absolute frequencies and proportions, whereas continuous variables are presented as means and standard deviations or as medians with interquartile ranges.

The Kolmogorov-Smirnov test was used in order to assess whether the data were normally distributed. We then performed a bivariate analysis, using Pearson's chi-square or Fisher's exact test for categorical variables and the Shapiro-Wilk test or Mann-Whitney U test for continuous variables. Factors showing a significant association (p < 0.05) were selected for inclusion in a multiple logistic regression model.

## Results

Of the 58 deaths evaluated, 25 (43.1%) occurred during the day (between 6:00 and 18:00). Among the causes of death listed on the death certificates, there was a predominance of respiratory disorders, which were listed in 35 cases (60.3%), "unspecified respiratory failure" and "asthma attack" accounting for 12 (34.3%) and 6 (17.1%), respectively. Cardiovascular events and disorders of the digestive tract were also listed as causes of death in considerable proportions (Table 1).

**Table 1:** 1806-3713-jbpneu-40-04-00364

Characteristic	n = 58
Cause of death	
Respiratory disease	35 (60.3)
Cardiovascular disease	8 (13.8)
Disease of the digestive tract	4 (6.9)
Other	9 (15.5)
No data	2 (3.5)
Place of death	
Hospital	37 (63.8)
Emergency room	4 (6.9)
Outpatient clinic	4 (6.9)
Unspecified treatment center	5 (8.7)
Home	2 (3.4)
Public space	2 (3.4)
No data	4 (6.9)

a Values expressed as n (%).

In the sample as a whole, the majority of the patients were unemployed and had been born in the interior (rural part) of the state of Bahia (Table 2). In our analysis of the clinical characteristics of the sample (Table 3), we observed that the patients in the study group (those who died) had been followed at the ProAR Central Referral Outpatient Clinic for a shorter time than had the control patients. In addition, the proportion of cases of controlled asthma and the rate of adherence to the standard treatment were lower in the study group. 

**Table 2:** Sociodemographic characteristics of 58 patients who evolved to death and 232 who did not among those with severe asthma treated at the Central Referral Outpatient Clinic of the Bahia State Asthma Control Program, in Salvador, Brazil, between 2002 and 2010.a

Characteristic	Cases of death	Control cases
Age^b^	62.2 ± 16.4	57.3 ± 14.0
Gender		
Male	33 (56.9)	47 (20.3)
Female	25 (43.1)	185 (79.7)
Level of education		
None	10 (17.2)	35 (15.1)
Elementary school	17 (29.3)	111 (47.8)
High school	10 (17.2)	52 (22.4)
College	1 (1.8)	11 (4.7)
No data	20 (34.5)	23 (10.0)
Employment status		
Unemployed	40 (69.0)	153 (65.9)
Employed	14 (24.1)	67 (28.9)
No data	4 (6.9)	12 (5.2)
Place of birth		
State capital	22 (37.9)	91 (39.2)
Other	32 (55.2)	122 (52.6)
No data	4 (6.9)	19 (8.2)

a Values expressed as n (%), except where otherwise indicated

b Values expressed as mean ± SD.

**Table 3:** Clinical characteristics of 58 patients who evolved to death and 232 who did not among those with severe asthma treated at the Central Referral Outpatient Clinic of the Bahia State Asthma Control Program, in Salvador, Brazil, between 2002 and 2010.a

Clinical characteristic	Cases of death	Control cases
Length of ProAR follow-up, years^b^	2 ± 2	6 ± 2
Asthma controlled^c^	9 (15.5)	126 (54.3)
Regular use of maintenance medication^c^	35 (60.3)	205 (88.4)
Exacerbation^c^	14 (24.1)	19 (8.2)
Number of emergency room visits^c,d^	3.0 (2.0-10.0)	2.0 (1.0-5.0)
Number of hospital admissions^c^	6 (10.3)	24 (10.3)
Number of pulses of oral corticosteroid^c,d^	2.0 (1.0-4.0)	1.0 (1.0-2.0)
Missed work/school^c^	1 (1.7)	13 (5.6)
Never-smoker^e^	22 (37.9)	145 (62.5)
Family history of asthma^e^	28 (48.3)	138 (59.5)
Duration of asthma, years^d,e^	30 (10-50)	24 (10-40)
Positive allergy test result^c^	17 (29.3)	109 (47.0)
Use of a single inhaled corticosteroid^c^	23 (39.7)	100 (43.1)
Use of a long-acting bronchodilator combined with an inhaled corticosteroid^c^	51 (87.9)	225 (97.0)
Use of a short-acting bronchodilator^c^	39 (67.2)	166 (71.6)

**ProAR:** : Programa para o Controle da Asma na Bahia (Bahia State Asthma Control Program)

a Values expressed as n (%), except where otherwise indicated

b Values expressed as mean ± SD

c Within the year preceding the death

d Values expressed as median (interquartile range)

e Information obtained at enrollment in the ProAR.

Pulmonary function parameters are described in Table 4. As can be seen, in the final evaluation (i.e., the last evaluation conducted before death occurred), the patients in the study group showed lower values of FEV_1_ and less reversibility after administration of a short-acting bronchodilator than did those in the control group.

**Table 4:** Lung function of 58 patients who evolved to death and 232 who did not among those with severe asthma treated at the Central Referral Outpatient Clinic of the Bahia State Asthma Control Program, in Salvador, Brazil, between 2002 and 2010.a

Lung function parameter	Cases of death	Control cases
Pre-BD FEV_1_ > 60% of predicted	7 (12.1)	114 (49.1)
Post-BD FVC, % of predicted^b^	69.65 ± 24.03	84.39 ± 17.35
Pre-BD FEV_1_, % of predicted^b^	43.36 ± 17.33	60.22 ± 19.66
Post-BD FEV_1_, % of predicted^b^	48.02 ± 19.53	67.62 ± 19.82

BD : bronchodilator

a Values expressed as n (%), except where otherwise indicated

b Values expressed as mean ± SD

In the bivariate analysis (Table 5), we observed significant differences between the study group and the control group. We identified a correlation between age and mortality, the majority of the deaths occurring in individuals over 50 years of age. In addition, 33 (56.9%) of the deaths occurred in males. As previously mentioned, the proportion of cases of controlled asthma and the rate of adherence to the standard treatment were lower in the study group than in the control group. 

The variables that were significantly associated with mortality were included in the multivariate analysis (Table 5). Failure to achieve good control of asthma remained a risk factor for mortality, not only in the analysis of mortality from respiratory causes but also in that of all-cause mortality. 

**Table 5:** Bivariate and multivariate analyses of risk factors for mortality among individuals with severe asthma.

Factor		Bivariate analysis			Multivariate analysis		
All-cause mortality (n = 58)	Mortality from respiratory causes (n = 35)	OR	95% CI	p	OR	95% CI	p
> 50 years of age		1.025	1.003-1.048	0.025	1.001	0.994-1.009	0.781
Male gender		5.196	2.824-9.564	< 0.001	5.392	2.373-12.254	< 0.001
Irregular use of maintenance medication in the last year		2.547	1.117-5.808	0.026	0.963	0.303-3.058	0.963
Uncontrolled asthma in the last year		5.338	2.443-11.665	< 0.001	2.796	1.135-6.890	0.025
FEV_1_ > 60% of predicted		0.953	0.934-0.972	< 0.001	0.176	0.057-0.539	0.002
	Male gender	3.850	1.782-8.314	0.001	4.550	1.499-13.814	0.007
	Uncontrolled asthma in the last year	8.089	2.850-22.955	< 0.001	3.448	1.035-11.487	0.044
	Asthma exacerbation in the last year	0.162	0.061-0.427	< 0.001	0.316	0.089 -1.115	0.073
	FEV_1_ > 60% of predicted	0.949	0.925-0.974	< 0.001	0.322	0.081-1.279	0.107

## Discussion

In the present study, most of the deaths among patients with severe asthma were attributed to asphyxiation, asthma attack and respiratory failure being the causes of death most often listed on the death certificates. The majority of the deaths occurred at health care facilities, hospitals predominating. Being male was found to increase the risk of death, as was non-adherence to asthma treatment and failure to achieve good asthma control. 

The principal objective of asthma treatment is to achieve symptom control and to reduce the risk of future complications of the disease. ^(^ 14 ^)^ Asthma control can be achieved through continuous use of the appropriate medication. Failure to control the symptoms of asthma can result in exacerbations and hospitalization, as well as, presumably, being associated with fatal outcomes.^(^ 2 ^)^

Most deaths from asthma are avoidable, because they represent adverse events resulting from poor asthma control, which is in turn related to factors that can be controlled^(^ 16 ^)^: failure to recognize the severity of a asthma attack and prescribe the appropriate treatment; the lack of a written asthma action plan; inappropriate emergency treatment; delayed hospital admission; and impeded access to health care services, essential medications, and treatment by health care professionals.

A lack of asthma control could be a risk factor for mortality among individuals with severe asthma. In addition to the severity of the disease *per se*, the level of asthma control can be negatively affected by patient denial or underestimation of the seriousness of the disease, the failure to use or the incorrect use of asthma medications, comorbidities, and poor patient perception of bronchial obstruction.^(^ 17 ^,^ 18 ^)^

The ProAR is designed to provide treatment, education, and investigation by a multidisciplinary team trained in the management of severe asthma, within the context of the SUS.^(^ 13 ^)^ One of the goals of the program is to furnish asthma medication, on a regular basis and at no charge, to patients with severe persistent asthma, in order to help such patients achieve and maintain good control of the disease. Patients followed at the ProAR Central Referral Outpatient Clinic receive guidance regarding and supervision of their use of inhaled medication.^(^ 13 ^)^

Our bivariate analysis revealed that, in relation to all-cause mortality, failure to use maintenance medication doubled the risk of death. However, that association did not remain significant in the multivariate analysis. 

An earlier study identified certain factors as being predictive of poor adherence to treatment among patients enrolled in the ProAR,^(^ 19 ^)^ including adverse events, great distances between the patient residence/workplace and the health care facility, transportation difficulties, and short dosage intervals for prescriptions involving multiple doses. Factors related to a lack of asthma control include non-adherence to treatment, a precipitous reduction in the dose of inhaled corticosteroid, carelessness in maintaining environmental controls, and comorbidities.^(^ 19 ^)^ In another ProAR study,^(^ 20 ^)^ correct inhaler technique was found to be associated with asthma symptom control. The authors suggested that the inhaler techniques employed by asthma patients should be evaluated, on a regular basis, by a multidisciplinary team.

A short duration of follow-up is another factor that might be associated with difficulty in achieving good asthma control. In many cases, prolonged treatment is needed in order to achieve such control. In the present study, the rate of good asthma control in the last year was only 15.5% among the patients who died. Although some of those patients had access to a well-trained multidisciplinary team of specialists and were treated with high doses of inhaled corticosteroids, combined with long-acting β_2_ agonists or other asthma medications, the evolution was unfavorable, resulting in death.

Of the deaths evaluated in the present study, the majority (63.8%) occurred in hospitals, which is in agreement with the findings of other such studies conducted in Brazil.^(^ 21 ^)^ We found that 44.6% of the deaths were attributed to asphyxiation. Asthma attacks and respiratory failure, the principal causes of death in the present study, could be related to a failure to recognize the severity of the asthma exacerbation, to a failure to follow the asthma action plan prescribed, or to delays in the initiation of treatment.

A portion of asthma-related deaths result from severe, fulminant exacerbations. The reasons why asthma patients who die tend to do so in hospitals have yet to be clarified, although characteristics of the airway obstruction itself, infections, and other comorbidities could play a role. In emergency rooms, unfavorable outcomes are associated with delays in treatment, difficulty in recognizing the warning signs of asthma exacerbation, and a lack of simplified protocols for the management of such exacerbations.^(^ 16 ^)^ Poor patient perception of the degree of bronchial obstruction is another, subjacent cause of fatal exacerbations. Asthma patients with a limited perception of their disease are at a greater risk of underestimating it and therefore receiving inadequate treatment.^(^ 17 ^,^ 22 ^)^

Asthma continues to be neglected or underestimated by governments, health care professionals, and patients. Efficient public policies and equitable access to asthma treatment could reduce the morbidity and mortality associated with the disease. In Brazil, no nationwide asthma control plan has yet been implemented. Some isolated initiatives have been quite successful, one example being the ProAR, the goal of which is to coordinate activities related to the prevention of and treatment of patients with severe asthma, conducted within the context of the SUS in the state of Bahia. Via the ProAR, asthma patients receive the necessary medication and are followed by a multidisciplinary health care team, as well as being exposed to asthma training and education, the ultimate objective being to achieve and maintain good asthma control.^(^ 13 ^)^

In our sample, being male was a risk factor for mortality. The protective effect of being female might be explained certain differences between men and women: women seek treatment more often than do men, who tend to seek treatment only when their symptoms are severe; the rate of adherence to treatment for chronic diseases is higher in women; and the severity of such diseases tends to be greater in men.^(^ 21 ^-^ 25 ^)^

Asthma is a chronic inflammatory disease that can progress to partial bronchial remodeling, together with the destruction of the airways and parenchyma, leading to a progressive decline in lung function, the degree of which depends on the duration of asthma and the age of the patient.^(^ 26 ^)^ The risk of asthma-related mortality increases in parallel with advancing age.^(^ 6 ^,^ 9 ^)^ In our bivariate analysis, we identified a borderline association between advanced age and all-cause mortality, although that association did not remain significant in the multivariate analysis. Most of the deaths evaluated in the present study occurred in individuals over 50 years of age with long-standing asthma and lung function impairment greater than that observed in their younger counterparts. 

Indicators of airflow limitation are also powerful predictors of mortality in patients with asthma. ^(^ 27 ^,^ 28 ^)^ Objective measures of lung function, such as PEF and FEV_1_, are useful predictors of hospital admission in such patients.^(^ 29 ^)^ In the present study, the patients who eventually died had presented worse lung function than had those who did not. A decline in FEV_1_ has been shown to be associated with death from asthma.^(^ 7 ^,^ 30 ^)^ Among the patients with severe asthma evaluated here, the risk of death was lower in those with better FEV_1_ values. Among those who died, shorter ProAR follow-up periods might have limited any potential gains in lung function.

Our study has certain limitations. The results cannot necessarily be generalized to patients with moderate or mild asthma. Other limitations include the retrospective study design and the general unreliability of data collected from death certificates. However, the data related to the deaths evaluated here were obtained from official documents that are used in order to track most of the health indicators in Brazil. The relevance of the present study lies in the information its provides regarding characteristics of the deaths that occurred within a sample of patients with severe asthma. In addition, the retrospective nature of our study is counterbalanced by the fact that the case-control study was nested within a specific cohort, which increases the quantity and quality of the data available for analysis. To our knowledge, this was the first controlled study to evaluate the risk factors for asthma-related mortality by systematic collection of data related to multiple clinical and functional variables. We are also unaware of any previous controlled studies showing a statistically significant association between uncontrolled asthma and mortality. 

Knowledge of the risk factors for asthma-related mortality is crucial to the planning and provision of individualized treatment of the disease and, consequently, to reducing the associated morbidity and mortality. Most asthma-related deaths could be avoided through early diagnosis and timely treatment of the disease, as well as improved training of health care teams, educational interventions to instruct patients in asthma self-management, and asthma-control programs.

The statistical power of our sample is limited by the relatively low number of deaths occurring within the period studied. Although we attempted to investigate all relevant deaths and employed four control cases for every case of death, the statistical power was still insufficient to make precise inferences of associations between asthma-related mortality and any of the numerous variables evaluated. However, the lack of a statistical association does not exclude the possibility that a given variable is a risk factor for mortality. Despite the low statistical power, we identified certain significant associations, which should therefore be given even more weight. 

In conclusion, most of the deaths evaluated in the present study were attributed to respiratory disorders and occurred in a hospital. Uncontrolled asthma, FEV_1_ pre-bronchodilator < 60% of predicted, and male gender were found to be significantly and independently associated with mortality among patients with severe asthma. 
