# Supplementary material for: Risk factors for death in patients with severe asthma
Source: J Bras Pneumol. 2014 Jul-Aug;40(4):364–72. doi: 10.1590/S1806-37132014000400003 (PMC4201166; doi:10.1590/S1806-37132014000400003)
Supplement: Translation

# Translation: Fatores de risco de morte em pacientes portadores de asma grave*

**Authors:** Andréia Guedes Oliva Fernandes, Carolina Souza-Machado, Renata Conceição Pereira Coelho, Priscila Abreu Franco, Renata Miranda Esquivel, Adelmir Souza-Machado, Álvaro Augusto Cruz

**Affiliations:** Programa para o Controle da Asma na Bahia - ProAR - Salvador (BA) Brasil; Escola de Enfermagem, Universidade Federal da Bahia, Salvador (BA) Brasil; Faculdade de Medicina, Universidade Federal da Bahia, Salvador (BA) Brasil; Escola Bahiana de Medicina e Saúde Pública, Salvador (BA) Brasil; Núcleo de Excelência em Asma, Universidade Federal da Bahia, Salvador (BA) Brasil; Instituto de Ciências da Saúde, Universidade Federal da Bahia; e Coordenador. Programa para o Controle da Asma na Bahia - ProAR - Salvador (BA) Brasil; Faculdade de Medicina, Universidade Federal da Bahia, Salvador (BA) Brasil

**Keywords:** Asma/mortalidade, Asma/terapia, Fatores de risco

## Abstract

**OBJETIVO:**

Identificar os fatores de risco para morte em pacientes com asma grave.

**MÉTODOS:**

Estudo caso-controle aninhado a uma coorte de pacientes acompanhados no Ambulatório Central de Referência do Programa para o Controle da Asma na Bahia, em Salvador (BA). No período entre dezembro de 2002 e dezembro de 2010, foram selecionados todos os pacientes com asma grave que foram a óbito e pacientes asmáticos graves vivos como controles na relação 1:4. As informações foram coletadas nos prontuários do serviço e complementadas por meio de visitas domiciliares e atestados de óbitos.

**RESULTADOS:**

Foram selecionados 58 óbitos e 232 controles. Os óbitos, na sua maioria, foram atribuídos a causas respiratórias e ocorreram dentro de uma unidade de saúde. Idade avançada, inatividade laboral, presença de rinite, sintomas de doença do refluxo gastroesofágico, tempo prolongado de doença e obstrução ao fluxo aéreo persistente foram aspectos comuns em ambos os grupos. A análise multivariada mostrou que o gênero masculino, VEF_1_ pré-broncodilatador < 60% do previsto e a ausência de controle dos sintomas da asma foram fatores de risco significativamente e independentemente associados à mortalidade nessa amostra de asmáticos graves.

**CONCLUSÕES:**

Nesta coorte ambulatorial de pacientes com asma grave, os óbitos ocorreram predominantemente por causas respiratórias em unidades de saúde. A falta de controle da asma e o gênero masculino foram os fatores de risco para óbito.

## Introdução

A asma é uma doença respiratória crônica comum que causa substanciais impactos na morbidade e mortalidade da população mundial. Estima-se que 10% dos asmáticos apresentam a forma grave da doença, causando impactos econômicos e sociais negativos e resultando em um ônus desproporcional na utilização dos serviços de saúde, na redução da qualidade de vida e no sofrimento humano imensurável devido à asfixia recorrente.^(^ 1 ^,^ 2 ^)^

A mortalidade por asma não progride em paralelo com sua prevalência.^(^ 1 ^)^ Tem sido observado que os países que apresentaram uma redução ou estabilização das mortes por asma foram aqueles que adotaram algumas estratégias voltadas para o controle da doença, tais como: i) diagnóstico precoce^(^ 3 ^,^ 4 ^)^; ii) disponibilidade de tratamento na atenção básica^(^ 4 ^-^ 6 ^)^; iii) fácil acesso aos serviços de saúde^(^ 5 ^,^ 7 ^)^; iv) atividades educativas direcionadas para o controle da enfermidade^(^ 3 ^,^ 4 ^)^; e v) capacitação adequada dos profissionais de saúde.^(^ 3 ^)^

O melhor entendimento dos fatores associados aos óbitos por asma permitirá uma melhor definição das estratégias preventivas para evitar eventos fatais.^(^ 8 ^)^ Os fatores de risco atribuídos às mortes por asma são os seguintes: a maior gravidade da doença^(^ 7 ^,^ 9 ^)^; a descontinuidade dos cuidados médicos^(^ 8 ^,^ 10 ^)^; situações socioeconômicas e psicossociais adversas^(^ 7 ^,^ 9 ^,^ 11 ^)^; e a conduta frente à doença (falta de acesso a terapia eficaz, má adesão à terapêutica e manejo inadequado da asma).

O objetivo do presente estudo foi identificar os fatores associados à mortalidade entre asmáticos graves em uma coorte ambulatorial com quase 10 anos de seguimento no Programa para o Controle da Asma na Bahia (ProAR), na cidade de Salvador (BA).^(^ 12 ^)^

## Métodos

### Desenho, amostra e local do estudo

Estudo de caso-controle aninhado a uma coorte, constituído por uma amostra de 58 óbitos ocorridos entre asmáticos graves e 232 controles vivos acompanhados na Central de Referência do ProAR no período entre dezembro de 2002 e dezembro de 2010. O estudo foi aprovado pelo comitê de ética e pesquisa institucional.

O ProAR tem como meta prioritária coordenar as ações de prevenção e assistência a pacientes com asma grave no âmbito do Sistema Único de Saúde (SUS). Os pacientes em acompanhamento pelo serviço, trimestralmente, têm acesso ao fornecimento de medicações gratuitas, consultas com uma equipe de saúde multidisciplinar e capacitada e sessões educativas em asma. As ações objetivam a obtenção e a manutenção do controle da asma.^(^ 13 ^)^

O diagnóstico e a classificação da gravidade da asma foram realizados por meio do levantamento dos sintomas e da medida do PFE realizado por um pneumologista no momento da admissão no serviço, seguindo os critérios da *Global Initiative for Asthma*.^(^ 14 ^)^ O controle da doença, por sua vez, foi obtido mediante a aplicação do *Asthma Control Questionnaire* com seis questões. Esse questionário inclui a avaliação de sintomas de asma e de uso de broncodilatadores de alívio nos últimos sete dias. Esse instrumento foi validado para língua portuguesa no nosso serviço e, naquele estudo, escores ≥ 1,5 foram aqueles que apresentaram melhor acurácia para identificar asma não controlada.^(^ 15 ^)^

### Identificação dos casos

Foram avaliados todos os registros de pacientes admitidos no ProAR, clinicamente diagnosticados como portadores de asma grave, que estavam em acompanhamento por uma equipe multiprofissional e em utilização de corticosteroide inalatório regularmente por, pelo menos, 3 meses e que progrediram para o óbito no período do estudo.

Os casos de óbitos foram identificados a partir dos registros em prontuários do ProAR para o período estudado. O registro é feito mediante o contato dos familiares com o serviço para informar sobre a ocorrência do óbito ou mediante a busca ativa daqueles pacientes que estivessem sem comparecer ao serviço por um período igual ou superior a 6 meses.

Foram identificados 62 óbitos, dos quais não foi possível obter informações completas em prontuário em 8 casos. Realizou-se, então, a visita domiciliar para esclarecimentos e busca das certidões de óbitos pendentes. Os resultados foram os seguintes: houve confirmação do óbito por familiares e vizinhos em 4 casos e, nos outros 4 casos, houve a impossibilidade de acesso às residências dos casos por estarem localizadas em áreas consideradas como de alto risco para crimes pelas comunidades vizinhas, sendo os pesquisadores desaconselhados a tentarem visitas aos domicílios.

As cópias dos atestados de óbitos foram arquivadas nos prontuários médicos dos pacientes. Para pacientes que não tinham uma cópia da declaração do óbito registrada, elaborou-se um ofício com a solicitação das respectivas informações (data, hora, local, causa básica e associada do óbito) direcionada à Diretoria de Informação em Saúde da Secretaria de Saúde da Bahia e à Subcoordenação de Informações em Saúde da Secretaria Municipal de Saúde de Salvador (BA).

### Identificação dos controles

Asmáticos graves vivos foram selecionados aleatoriamente através do programa Microsoft Excel 2010 a partir de um banco de dados de prontuários existente no ProAR.

### Extração dos dados

O grupo caso foi estratificado conforme as seguintes faixas etárias: 10-30 anos, 31-50 anos e acima de 50 anos. Cada controle sorteado foi alocado para o estrato correspondente à sua idade e posteriormente pareado pelo ano da última consulta em relação ao óbito. Para cada caso foram identificados 4 controles.

Os dados foram coletados a partir das informações obtidas durante as visitas domiciliares, nos atestados de óbitos e nos prontuários do serviço, constituídos por impressos estruturados obedecendo à seguinte organização: (a) ficha de acompanhamento da história clínica do paciente desde a última consulta; (b) consultas realizadas pelas equipes de enfermagem, equipe médica, psicólogos e assistentes sociais; (c) ficha de admissão do paciente ao serviço; (d) cópia dos exames realizados pelo paciente; e (e) registro de dispensação dos medicamentos realizados pela farmácia. O prontuário foi preenchido sistematicamente a cada consulta de rotina, realizada trimestralmente.

As variáveis analisadas foram as seguintes: sociodemográficas (idade, gênero, estado ocupacional, nível educacional e naturalidade); clínicas (tempo de admissão no programa, história de hospitalizações, visitas as salas de emergência, número de pulsos de corticoide oral (> 3 dias), número de exacerbações da asma, duração da doença e estado do controle dos sintomas da asma na última avaliação no ProAR); antecedentes familiares (história de asma na família); tabagismo; resultados dos exames (prova de função pulmonar e teste para diagnóstico de alergia); registros de dispensação da farmácia e de adesão ao tratamento; e informações sobre o óbito (data, hora, local e causas primárias e secundárias associadas ao óbito).

### Análise estatística

Os dados coletados foram analisados através do Statistical Package for the Social Sciences, versão 17.0 (SPSS Inc., Chicago, IL, EUA). As variáveis categóricas foram analisadas conforme a sua frequência absoluta simples e em proporção. As variáveis contínuas foram analisadas através de média, mediana, desvio-padrão e amplitude interquartílica.

Após a realização do teste de Kolmogorov-Smirnov para a avaliação da normalidade da distribuição dos dados, foi realizada a análise bivariada através dos testes do qui-quadrado de Pearson ou exato de Fisher (variáveis categóricas) e os testes de Shapiro-Wilk ou U de Mann-Whitney (variáveis contínuas). Os fatores associados aos óbitos por asma com nível descritivo inferior a 0,05 (p < 0,05) foram considerados significativos e inseridos no modelo de regressão logística múltipla.

## Resultados

Entre os 58 óbitos com dados disponíveis para avaliação, observou-se que 25 (43,1%) das mortes ocorreram no período diurno (6-18 h). Entre as causas registradas nos atestados de óbito, houve predominância das causas respiratórias, em 35 casos (60,3%), sendo decorrentes de insuficiência respiratória não especificada, em 12 (34,3%), e de crise asmática, em 6 (17,1%). Causas cardiovasculares e causas ligadas ao aparelho digestivo também foram registradas em proporções consideráveis (Tabela 1).

**Tabela 1:** Caracterização geral de 58 pacientes com asma grave que foram a óbito da coorte do Ambulatório Central de Referência do Programa para o Controle da Asma na Bahia entre 2002 e 2010.a

Características	Casos óbitos
Causa do óbito	
Respiratória	35 (60,3)
Cardiovascular	8 (13,8)
Ligada ao aparelho digestivo	4 (6,9)
Outras causas	9 (15,5)
Sem informação	2 (3,5)
Local do óbito	
Hospital	37 (63,8)
Pronto-socorro ou pronto-atendimento	4 (6,9)
Ambulatório	4 (6,9)
Unidade sem especificação	5 (8,7)
Residência	2 (3,4)
Via pública	2 (3,4)
Sem informação	4 (6,9)

a Valores expressos em n (%).

Quanto à caracterização sociodemográfica, os pacientes de ambos os grupos, na sua maioria, apresentavam inatividade laboral e eram naturais do interior do estado da Bahia (Tabela 2). Na análise das características clínicas (Tabela 3), observou-se que o grupo caso apresentava menor tempo de acompanhamento no ambulatório do ProAR, assim como menor proporção de asma controlada e de adesão ao tratamento regular.

**Tabela 2:** Características sociodemográficas dos 58 pacientes com asma grave que foram a óbito e dos 232 controles vivos com asma grave acompanhados no Programa para o Controle da Asma na Bahia entre 2002 e 2010.a

Características	Casos óbitos	Controles vivos
Idade^b^	62,2 ± 16,4	57,3 ± 14,0
Gênero		
Masculino	33 (56,9)	47 (20,3)
Feminino	25 (43,1)	185 (79,7)
Nível educacional		
Não alfabetizado	10 (17,2)	35 (15,1)
Ensino fundamental	17 (29,3)	111 (47,8)
Ensino médio	10 (17,2)	52 (22,4)
Ensino superior	1 (1,8)	11 (4,7)
Sem informação	20 (34,5)	23 (10,0)
Estado ocupacional		
Inativo	40 (69,0)	153 (65,9)
Ativo	14 (24,1)	67 (28,9)
Sem informação	4 (6,9)	12 (5,2)
Naturalidade		
Capital	22 (37,9)	91 (39,2)
Interior	32 (55,2)	122 (52,6)
Sem informação	4 (6,9)	19 (8,2)

a Valores expressos em n (%), exceto onde indicado

b Valores expressos em média ± dp.

**Tabela 3:** Características clínicas dos 58 pacientes com asma grave que foram a óbito e dos 232 controles vivos com asma grave acompanhados no Programa para o Controle da Asma na Bahia (ProAR) entre 2002 e 2010.a

Características clínicas	Casos óbitos	Controles vivos
Tempo de acompanhamento no ProAR, anos^b^	2 ± 2	6 ± 2
Asma controlada^c^	9 (15,5)	126 (54,3)
Uso regular da medicação de controle^c^	35 (60,3)	205 (88,4)
Presença de exacerbação^c^	14 (24,1)	19 (8,2)
Número de idas à emergência^c,d^	3,0 (2,0-10,0)	2,0 (1,0-5,0)
História de internação^c^	6 (10,3)	24 (10,3)
Ciclos de corticoide oral^c,d^	2,0 (1,0-4,0)	1,0 (1,0-2,0)
Ausência no trabalho/escola^c^	1 (1,7)	13 (5,6)
Nunca fumou^e^	22 (37,9)	145 (62,5)
História familiar de asma^e^	28 (48,3)	138 (59,5)
Duração da asma, anos^d,e^	30 (10-50)	24 (10-40)
Teste alérgico positivo^c^	17 (29,3)	109 (47,0)
Uso de corticoide inalatório em dispositivo onde não há combinação^c^	23 (39,7)	100 (43,1)
Uso de broncodilatador de longa ação associado com corticoide inalatório^c^	51 (87,9)	225 (97,0)
Uso de broncodilatador de curta ação^c^	39 (67,2)	166 (71,6)

a Valores expressos em n (%), exceto onde indicado

b Valores expressos em média ± dp

c Informação do último ano em relação ao óbito

d Valores expressos em mediana (amplitude interquartílica)

e Informação obtida durante a consulta de admissão ao serviço.

Os parâmetros da função pulmonar estão descritos na Tabela 4. Pode-se observar que o grupo caso, na última avaliação antes do óbito, apresentou VEF_1_ mais reduzido e menor reversibilidade com broncodilatador de curta ação, em comparação ao grupo controle.

**Tabela 4:** Caracterização da função pulmonar dos 58 pacientes com asma grave que foram a óbito e dos 232 controles vivos com asma grave acompanhados no Programa para o Controle da Asma na Bahia (ProAR) entre 2002 e 2010.a

Características da função pulmonar	Casos óbitos	Controles vivos
VEF_1 _pré-BD > 60%	7 (12,1)	114 (49,1)
% CVF pós-BD^b^	69,65 ± 24,03	84,39 ± 17,35
% VEF_1 _pré-BD^b^	43,36 ± 17,33	60,22 ± 19,66
% VEF_1 _pós-BD^b^	48,02 ± 19,53	67,62 ± 19,82

BD : broncodilatador

a Valores expressos em n (%), exceto onde indicado

b Valores expressos em média ± dp.

Na análise bivariada (Tabela 5), observaram-se diferenças significativas entre os casos (óbitos) e os controles asmáticos graves vivos. Houve uma correlação entre mortalidade e idade. A maioria das mortes ocorreu entre indivíduos com mais de 50 anos. Além disso, grande parte das mortes ocorreu em pacientes do gênero masculino, em 33 casos (56,9%). O grupo caso apresentou menor proporção de asma controlada e menor adesão ao tratamento regular. 

**Tabela 5:** Análises bivariadas e multivariadas de fatores potencialmente associados à mortalidade entre asmáticos graves.

Fatores		Análise bivariada			Análise multivariada		
Óbitos por todas as causas (n = 58)	Causas respiratórias (n = 35)	OR	IC95%	p	OR	IC95%	p
Idade > 50 anos		1,025	1,003-1,048	0,025	1,001	0,994-1,009	0,781
Gênero masculino		5,196	2,824-9,564	< 0,001	5,392	2,373-12,254	< 0,001
Uso irregular da medicação de controle no último ano		2,547	1,117-5,808	0,026	0,963	0,303-3,058	0,963
Asma não controlada no último ano		5,338	2,443-11,665	< 0,001	2,796	1,135-6,890	0,025
VEF_1 _pré-broncodilatador > 60% do previsto		0,953	0,934-0,972	< 0,001	0,176	0,057-0,539	0,002
	Gênero masculino	3,850	1,782-8,314	0,001	4,550	1,499-13,814	0,007
	Asma não controlada no último ano	8,089	2,850-22,955	< 0,001	3,448	1,035-11,487	0,044
	Exacerbação da asma no último ano	0,162	0,061-0,427	< 0,001	0,316	0,089 -1,115	0,073
	VEF_1 _pré-broncodilatador > 60% do previsto	0,949	0,925-0,974	< 0,001	0,322	0,081-1,279	0,107

As variáveis significativamente associadas à mortalidade entre asmáticos graves foram incluídas na análise multivariada (Tabela 5). A ausência do controle da asma permaneceu como um fator de risco relacionado ao óbito tanto na análise por todas as causas de óbitos, como na análise por causas respiratórias.

## Discussão

No presente estudo, observou-se que os óbitos entre asmáticos graves ocorreram principalmente por asfixia, sendo crise asmática e insuficiência respiratória as principais causas registradas nos atestados de óbito. Em sua maioria, os óbitos ocorreram em serviços de saúde, predominantemente em hospitais. O risco de morte foi mais elevado para homens, para pacientes em tratamento irregular da asma e para pacientes com asma grave não controlada.

O principal objetivo do tratamento da asma é a obtenção do controle dos sintomas e a redução de riscos futuros decorrentes da doença.^(^ 14 ^)^ O controle da asma pode ser obtido através do uso adequado de medicamentos em tratamento contínuo. A ausência de controle dos sintomas da asma pode resultar em exacerbações e em hospitalizações, e supõe-se estar associada a desfechos fatais.^(^ 2 ^)^

Óbitos por asma são na maioria das vezes evitáveis, já que são eventos decorrentes do controle inadequado da doença e relacionados aos seguintes fatores adicionais: falhas na identificação da gravidade das crises e na prescrição da terapêutica apropriada; inexistência de um plano de ação escrito; tratamento emergencial inadequado; retardo nas internações; e dificuldade de acesso a cuidados de saúde, medicamentos essenciais e profissionais de saúde.^(^ 16 ^)^

A ausência de controle da asma pode ser um fator de risco associado ao óbito entre asmáticos graves. De forma geral, além da própria gravidade da doença, a negação ou subestimação da doença pelo paciente, a falta de utilização ou a utilização incorreta das medicações antiasmáticas, a presença de comorbidades e a má percepção da obstrução brônquica podem influenciar no controle dos sintomas da asma.^(^ 17 ^,^ 18 ^)^

O ProAR é um projeto de assistência, ensino e pesquisa constituído por uma equipe multiprofissional capacitada na prestação de um atendimento global ao portador de asma grave no âmbito do SUS.^(^ 13 ^)^

O ProAR tem como uma das suas metas fornecer regularmente medicamentos gratuitos aos portadores de asma persistente grave para a obtenção e a manutenção do controle da doença. Os pacientes em acompanhamento pelo serviço recebem orientações e são supervisionados quanto à utilização da medicação inalatória.^(^ 13 ^)^ Foi evidenciado pela análise bivariada, em relação a todas as causas de óbitos, que aqueles que não utilizavam regularmente a medicação de controle apresentavam um risco aumentado em mais de duas vezes de evoluir ao óbito. Entretanto, essa associação perdeu a significância estatística na análise multivariada.

Alguns fatores preditores para a má adesão ao tratamento foram identificados entre os pacientes acompanhados pelo serviço em um estudo prévio,^(^ 19 ^)^ tais como a presença de eventos adversos, a distância da residência/local de trabalho da unidade de atendimento, a dificuldade de transporte e o intervalo posológico nas prescrições com doses múltiplas. A falta de adesão ao tratamento regular, a redução precipitada da dose do corticoide inalatório, a falta de cuidado com o controle ambiental e a existência de comorbidades podem estar relacionadas com a falta de controle da asma. ^(^ 19 ^)^ Outro fator identificado no ProAR que está associado ao controle dos sintomas é a utilização da técnica inalatória adequada. A técnica de inalação incorreta é um dos fatores que podem dificultar o controle da asma e deve ser avaliada pela equipe multiprofissional regularmente.^(^ 20 ^)^

O tempo de acompanhamento no serviço é outro fator que pode estar relacionado com a dificuldade na obtenção do controle da asma. Em muitos casos, é preciso algum tempo para que o controle da asma seja obtido com o tratamento. No presente estudo, foi constatada uma proporção de 15,5% de asma controlada no último ano entre os casos de óbitos. Apesar de esse subgrupo de pacientes ter acesso a uma equipe multidisciplinar capacitada e especializada e utilizar corticoides inalatórios em doses elevadas, combinados a β_2_-agonistas de longa duração e/ou outros medicamentos antiasmáticos, ainda assim esse subgrupo evoluiu desfavoravelmente para o óbito.

Identificou-se que a maioria dos óbitos ocorreu em hospitais (63,8%), corroborando resultados de outros autores no Brasil.^(^ 21 ^)^ Identificou-se que 44,6% dos óbitos do presente estudo foram atribuídos à asfixia. As mortes por asma e insuficiência respiratória, principais causas de óbitos no presente estudo, podem estar relacionadas à incapacidade no reconhecimento da gravidade da exacerbação da doença, à falta de seguimento do plano de ação prescrito e ao retardo no tratamento.

Uma parcela dos óbitos relacionados à asma é decorrente de exacerbações graves e fulminantes; as razões pelas quais os pacientes asmáticos morrem em hospitais no nosso meio ainda não estão esclarecidas. Características da própria obstrução das vias aéreas, infecções e comorbidades podem contribuir para isso. Em unidades de emergência, a evolução desfavorável está associada ao retardo na intervenção terapêutica, à dificuldade no reconhecimento dos sinais de alerta para uma exacerbação da asma e à ausência de protocolos simplificados de ação.^(^ 16 ^)^ A má percepção da gravidade da obstrução brônquica é outra causa subjacente de exacerbações fatais em asmáticos. Asmáticos com má percepção de sua doença têm maior risco de subestimá-la e de receber tratamento insuficiente.^(^ 17 ^,^ 22 ^)^

A asma continua a ser negligenciada por governantes, profissionais de saúde e pacientes. Esforços governamentais para oferecer políticas públicas eficientes e acesso equânime para o tratamento da asma podem concorrer para redução da morbidade e mortalidade pela doença. No Brasil, ainda não foi implementado um plano de abrangência nacional para o controle da asma. Observam-se iniciativas isoladas e bem sucedidas, a exemplo do ProAR, o qual tem por objetivo integrar ações de prevenção e assistência aos pacientes com asma grave no âmbito do SUS na Bahia, objetivando assegurar o fornecimento de medicamentos antiasmáticos, o acompanhamento por uma equipe multiprofissional e o oferecimento de atividades educativas a fim de obter e manter o controle da doença.^(^ 13 ^)^

Na presente amostra, ser do gênero masculino foi um fator de risco para a ocorrência das mortes. Fatores relacionados à procura pelos serviços de saúde, à aderência ao tratamento em portadores de doença crônica, à existência e a maior gravidade de comorbidades nos homens podem explicitar o efeito protetor no gênero feminino.^(^ 21 ^,^ 23 ^)^ Mulheres buscam com maior frequência os serviços de saúde, ao contrário dos homens, que tendem a recorrer às unidades apenas em casos graves.^(^ 24 ^,^ 25 ^)^

A asma é uma enfermidade inflamatória crônica que pode evoluir com remodelamento brônquico imperfeito e destruição das vias aéreas e do parênquima com perda progressiva da função pulmonar, sendo agravada pela duração da asma e pela idade do paciente.^(^ 26 ^)^ O risco de mortalidade por asma aumenta com a idade.^(^ 6 ^,^ 9 ^)^ No presente estudo, foi encontrada uma leve associação entre idade avançada e morte por todas as causas na análise bivariada. Todavia, essa associação desapareceu na análise multivariada. Os óbitos, na sua maioria, ocorreram em indivíduos com idade acima de 50 anos, que apresentavam tempo prolongado de doença e pior função pulmonar.

Indicadores de limitação ao fluxo aéreo constituem-se em fortes preditores de mortalidade entre asmáticos.^(^ 27 ^,^ 28 ^)^ Medidas objetivas da função pulmonar, tais como PFE e VEF_1_, são úteis como preditoras para a admissão hospitalar em pacientes asmáticos.^(^ 29 ^)^ Os indivíduos do estudo que evoluíram para o óbito apresentaram pior função pulmonar. Existe também uma associação entre o declínio do VEF_1_ e a mortalidade por asma.^(^ 7 ^,^ 30 ^)^ Os pacientes asmáticos graves estudados com melhor VEF_1_ apresentaram menor risco de morte. Um menor tempo de acompanhamento no ProAR pode ter limitado o potencial de ganho de função pulmonar no grupo dos pacientes que foram a óbito.

O presente estudo apresenta algumas limitações. Os resultados da presente pesquisa não podem ser assumidos como necessariamente válidos para pacientes com asma leve e moderada. Outros fatores relevantes são o seu caráter retrospectivo e a imprecisão no preenchimento das certidões de óbitos. Entretanto, as informações acerca dos óbitos no presente estudo foram extraídas de documentos oficiais que são utilizados como subsídio para a grande maioria dos indicadores de saúde no país. Ademais, devemos apontar a relevância do presente estudo no que se refere à obtenção de informações referentes à caracterização dos óbitos ocorridos numa coorte ambulatorial de indivíduos portadores de asma grave. Por outro lado, o estudo caso-controle, cujos resultados estão apresentados aqui, está aninhado numa coorte, o que aumenta muito o volume e a qualidade das informações disponíveis, não obstante o caráter retrospectivo da obtenção de dados para a análise. Não temos conhecimento de um estudo para a avaliação de fatores de risco para morte por asma que tenha contado com tantas variáveis clínicas e funcionais colhidas de forma sistemática e comparável entre os casos e os controles. Não temos conhecimento tampouco da existência de um estudo controlado que tenha confirmado de forma estatisticamente significante a associação entre a falta de controle da asma e a mortalidade por essa enfermidade crônica.

O conhecimento dos fatores associados à ocorrência do desfecho fatal é crucial para planejar e oferecer um atendimento personalizado ao indivíduo e, consequentemente, obter a redução da morbidade e mortalidade relacionadas à doença. As mortes por asma podem ser, na sua maioria, evitáveis se a doença for diagnosticada e tratada precocemente. A adoção de estratégias, tais como a capacitação das equipes de saúde, intervenções educativas para o automanejo da doença e a criação de programas voltados para o controle da asma, pode contribuir significativamente na redução da mortalidade pela doença.

O poder da amostra do presente estudo é limitado pelo número de óbitos que ocorreu no período de tempo avaliado. Embora tenhamos buscado investigar todos os óbitos e usado o recurso de quatro controles para cada caso, não há um poder estatístico suficiente para inferências precisas de associações entre as diversas variáveis do estudo e o risco de morte entre pessoas com asma. Logo, a ausência de associação estatisticamente significante na nossa análise não exclui a possibilidade de que uma determinada variável seja um fator associado à morte. Por outro lado, as associações significantes encontradas devem ser ainda mais valorizadas.

Em conclusão, os óbitos ocorreram predominantemente por causas respiratórias, em ambiente hospitalar. A falta de controle da asma, VEF_1_ pré-broncodilatador < 60% do previsto e o gênero masculino foram os fatores de risco associados significativamente e independentemente à ocorrência das mortes nessa coorte ambulatorial de pacientes com asma grave.
